# Major bleeding in patients with atrial fibrillation treated with apixaban versus warfarin in combination with amiodarone: nationwide cohort study

**DOI:** 10.1136/openhrt-2023-002555

**Published:** 2024-03-01

**Authors:** Astrid Fritz Hansson, Angelo Modica, Henrik Renlund, Christina Christersson, Claes Held, Gorav Batra

**Affiliations:** 1 Department of Medical Sciences Cardiology, Uppsala University, Uppsala, Sweden; 2 Pfizer AB, Stockholm, Sweden; 3 Uppsala Clinical Research Center, Uppsala University, Uppsala, Sweden

**Keywords:** atrial fibrillation, pharmacology, clinical, epidemiology

## Abstract

**Background:**

Amiodarone is an established treatment for atrial fibrillation (AF) but might interfere with the metabolism of apixaban or warfarin. Therefore, the aim was to investigate the occurrence of major bleeding among patients with AF treated with amiodarone in combination with apixaban or warfarin.

**Methods:**

Retrospective observational study using Swedish health registers. All patients with AF in the National Patient Register and the National Dispensed Drug Register with concomitant use of amiodarone and warfarin or apixaban between 1 June 2013 and 31 December 2018 were included. Propensity score matching was performed, and matched cohorts were compared using Cox proportional HRs. The primary outcome was major bleeding resulting in hospitalisation based on International Classification of Diseases (ICD)-10 codes. Secondary outcomes included intracranial bleeding, gastrointestinal bleeding and other bleeding. Exploratory outcomes included ischaemic stroke/systemic embolism and all-cause/cardiovascular (CV) mortality.

**Results:**

A total of 12 103 patients met the inclusion criteria and 8686 patients were included after propensity score matching. Rates of major bleeding were similar in the apixaban (4.3/100 patient-years) and warfarin cohort (4.5/100 patient-years) (HR: 1.03; 95% CI: 0.76 to 1.39) during median follow-up of 4.4 months. Similar findings were observed for secondary outcomes including gastrointestinal bleeding and other bleeding, and exploratory outcomes including ischaemic stroke/systemic embolism and all-cause/CV mortality.

**Conclusions:**

Among patients treated with amiodarone in combination with apixaban or warfarin, major bleeding and thromboembolic events were rare and with no significant difference between the treatment groups.

**EUPAS registry number:**

EUPAS43681.

**WHAT IS ALREADY KNOWN ON THIS TOPIC**

- Direct oral anticoagulants, such as apixaban, are often used for stroke prevention in patients with atrial fibrillation (AF). Additionally, antiarrhythmic drugs, including amiodarone, are frequently used in patients with AF. There is potential for interaction between amiodarone and apixaban, leading to concerns about an increased risk of major bleeding when both drugs are combined.

**WHAT THIS STUDY ADDS**

- In this nationwide cohort study using Swedish health registers, we included 8686 patients with AF who were treated with amiodarone, paired either with apixaban or warfarin. After propensity score matching, we found that apixaban+amiodarone was associated with a similar risk of major bleeding as warfarin+amiodarone. This finding was also observed for other secondary outcomes, including gastrointestinal bleeding and other types of bleeding. For intracranial bleeding, apixaban combined with amiodarone was associated with a lower risk compared with the combination of warfarin and amiodarone.

**HOW THIS STUDY MIGHT AFFECT RESEARCH, PRACTICE OR POLICY**

- In patients with AF treated with amiodarone, oral anticoagulation with apixaban or warfarin is equally as safe in terms of risk of major bleeding.

## Introduction

Atrial fibrillation (AF) is the most common sustained cardiac arrhythmia in adults worldwide.^1^ In patients with AF, apixaban has shown superiority to warfarin for stroke prevention and safety in terms of major bleeding, including intracranial bleeding.^2^ Antiarrhythmic drugs (AADs) are frequently used in patients with AF and with the primary indication being symptom control and improving health-related quality of life.^3^ Early initiation of rhythm control, mainly with AADs, has in recent studies also been associated with lower rates of cardiovascular (CV) events in patients with AF.^4^ Currently, the most effective drug for the prevention of recurrent episodes of AF among patients with paroxysmal or persistent AF is the class III AAD amiodarone.^5^

Amiodarone is metabolised in the liver and can affect the metabolism of numerous drugs, including warfarin and apixaban mainly through the cytochrome (CYP)2C9 and the CYP3A4 pathway, respectively.^6 7^ The interaction between amiodarone and warfarin decreases the time in therapeutic range (TTR) of the international normalised ratio (INR), thus, increasing the risk of adverse CV events.^8–10^ In a subgroup propensity-matched analysis of the Apixaban for Reduction in Stroke and Other Thromboembolic Events in Atrial Fibrillation (ARISTOTLE) trial (funded by Bristol Myers Squibb and Pfizer), amiodarone in combination with warfarin or apixaban was associated with an approximately 47% increased risk of stroke and systemic embolism compared with treatment with warfarin or apixaban alone.^10^ Furthermore, apixaban versus warfarin was associated with an approximately 26% lower rate of death and 39% lower rate of major bleeding when combined with amiodarone.^10^ However, randomised controlled trials often include selected patient populations that might not always be representative of real-life patients.^11^

Although the summary of product characteristics for apixaban mentions the risk of potentiation in apixaban plasma concentration when combined with amiodarone it is not deemed clinically important as amiodarone is not a strong inhibitor of both CYP3A4 and p-glycoprotein (P-gp). Therefore, the European Medical Agency (EMA) and the US Food and Drug Administration (FDA) state no contradiction against the combination of apixaban and amiodarone.^6 7^ As amiodarone is used to prevent recurrent episodes of paroxysmal or persistent AF, especially among patients with structural heart disease, it is important to understand the risks associated with combining it with apixaban and warfarin.^3^ Based on the paucity of real-life data, the objective of this observational cohort study was to investigate the real-world occurrence of major bleeding among patients treated with amiodarone in combination with apixaban or warfarin in unselected patients with AF.

## Methods

### Study population and data sources

This retrospective observational cohort study was based on data from various Swedish health registries: the National Patient Register, the National Dispensed Drug Register and the National Cause of Death Register. The National Patient Register is a mandatory registry containing primary and secondary diagnoses based on International Classification of Diseases (ICD) codes for all hospitalisations and outpatient visits in Sweden since 1987.^12^ The National Dispensed Drug Register contains data on all prescribed medications dispensed at pharmacies in Sweden since 2005.^13^ The National Cause of Death Register was established in 1961 and is a mandatory registry that contains mortality data including date and cause of death. The Swedish health registries provide complete national coverage and each of the registries has been highly validated.^12–14^ Data linkage between registries was performed by the National Board of Health and Welfare in Sweden using the 10-digit personal registration number available to all Swedish citizens. The current study was registered and approved by the Ethical Review Board in Sweden (application number 2021-04604).

**Figure 1:**
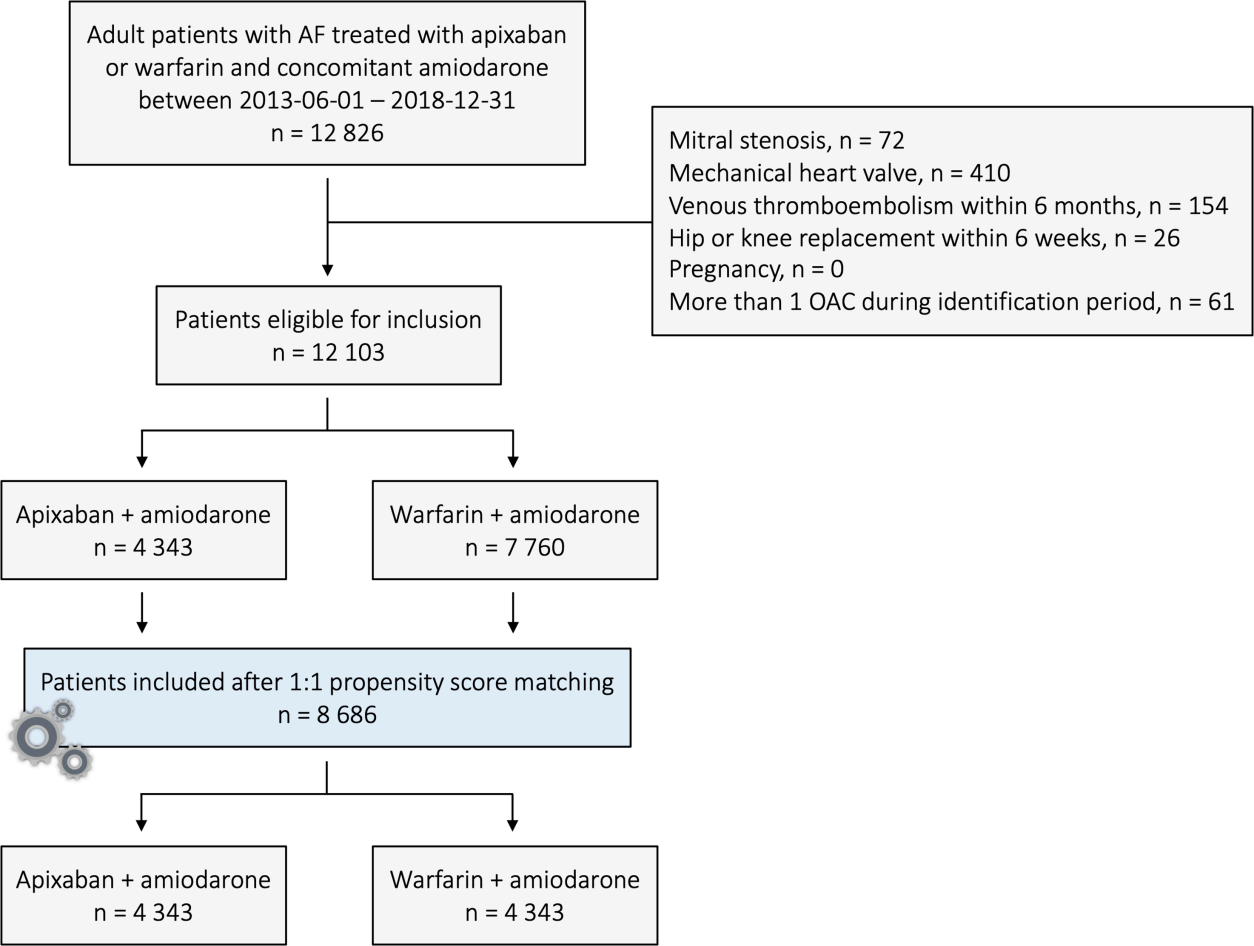
Consolidated Standards of Reporting Trials diagram. AF, atrial fibrillation; OAC, oral anticoagulants.

Patients registered with AF in the National Patient Register were included in the study if treated with amiodarone in combination with apixaban or warfarin according to the National Dispensed Drug Register. The study period was set between 1 June 2013 and 31 December 2018, marking the day of apixaban being approved for AF in Sweden and the end of available data, respectively. Patients registered in the National Patient Register with mitral stenosis, mechanical heart valve, acute venous thromboembolism within 6 months and/or with hip/knee replacement within 6 weeks prior to inclusion were excluded. Also, patients with ongoing pregnancy and those simultaneously receiving more than one oral anticoagulation were excluded (figure 1).

### Drug exposure

Exposure was defined at the index date as either amiodarone in combination with apixaban or amiodarone in combination with warfarin. The date of prescription of either apixaban or warfarin in patients already treated with amiodarone, or the date of prescription of amiodarone in patients already on apixaban or warfarin was set as the index date as this was when concomitant treatment began.

To establish on-treatment analysis, a pill consumption method was used for apixaban, calculating the number of pills dispensed and the number of pills expected to be consumed. A 30-day grace period was added to allow for some degree of non-compliance and irregular dispensing due to stockpiling. In contrast to the standardised doses of apixaban, the dosage of warfarin and amiodarone might vary between patients and over time. To establish warfarin exposure over time, dispense data combined with a mean dosage of warfarin/week based on age and sex from the Swedish oral anticoagulant registry (AuriculA) were used (online supplemental table 1). For amiodarone, each dispensation was estimated to last 3 months as drugs in Sweden cannot be prescribed in larger quantities than what is expected to last 3 months. A 30-day grace period was added when estimating warfarin and amiodarone exposure over time.

10.1136/openhrt-2023-002555.supp1 Supplementary data

### Covariates

Patient demographics and comorbidities were retrieved from the National Patient Register. Pharmacy-dispensed comedications dispensed within 6 months before index were obtained from the National Dispensed Drug Register (online supplemental table 2). Combined, this information formed the foundation for the baseline characteristics. There was no missing data about age, comorbidities and comedication. Missing data about sex for some patients (n=305) were imputed by random sampling from available values.

### Outcomes

Outcome data were obtained from National Patient Register and the Swedish Cause of Death Register using ICD-10 codes (online supplemental table 3). The primary outcome was major bleeding associated with hospitalisation, defined as fatal bleeding, intracranial bleeding, gastrointestinal bleeding or other major bleeding events (eg, pulmonary or urogenital bleedings). The ICD-10 codes corresponding to major bleeding have been validated with high sensitivity and specificity in Swedish patients with AF.^14^ Secondary outcomes were intracranial bleeding, gastrointestinal bleeding and other bleeding. Exploratory outcomes included ischaemic stroke/systemic embolism, all-cause mortality, and CV mortality.

### Statistics

Baseline characteristics were presented using frequencies and percentages for categorical variables and medians with IQR for continuous variables. Statistical differences between treatment groups before and after propensity score matching were tested using the Kruskal-Wallis or Pearson’s χ^2^ tests as appropriate.

Individual propensity scores for the likelihood of receiving apixaban+amiodarone rather than warfarin+amiodarone were obtained by logistic regression performed using the following covariates: age, sex, hypertension, diabetes mellitus, prior major bleeding, stroke/transient ischaemic attack/systemic embolism, myocardial infarction, percutaneous coronary intervention/coronary artery bypass graft surgery, heart failure, peripheral arterial disease, chronic kidney disease, liver disease, asthma, chronic obstructive pulmonary disease, cancer within 3 years, other antithrombotic drugs within preceding 6 months (aspirin, clopidogrel, ticagrelor, prasugrel, low-molecular-weight heparin) as well as oral anticoagulant (OAC) within 1 year. Optimal 1:1 matching was performed on the propensity score.

Kaplan-Meier estimates with accompanying at-risk tables for on-treatment analysis were plotted. Kaplan-Meier estimates were plotted for as long as there are at least 100 patients at risk in the smallest group. Matched cohorts were compared regarding outcome events using Cox regression analysis. Time at risk was counted from date of inclusion +1 day. Patients were censored at the time of outcome, death, drug change/discontinuation or end of follow-up.

In a supplementary analysis, and to minimise confounding by indication, only apixaban, warfarin and amiodarone-naive patients were included. In this analysis, patients with a dispense of any OACs within 1 year before index date were excluded. Additionally, we performed a subgroup analysis for the primary outcome (major bleeding) among patients aged <75 and ≥75 years.

All tests were two-sided with 95% CIs and with p values <0.05 considered as significant. All statistical analyses were performed at Uppsala Clinical Research Centre (UCR) with the use R V.4.2.1 (R Foundation for Statistical Computing).

## Results

### Patient characteristics and comorbidities

Among all patients diagnosed with AF as of 1 January 2018, in the National Patient Register, 1.1% were treated with amiodarone. After applying inclusion and exclusion criteria, 12 103 patients treated with amiodarone and concomitant warfarin or apixaban were included (4343 (35.9%) patients treated with apixaban and 7760 (64.1%) patients treated with warfarin) (figure 1). Patients receiving warfarin tended to be older, more often male and with higher occurrence of heart failure, myocardial infarction and ischaemic stroke than those treated with apixaban. Patients treated with apixaban had more often a history of cancer and prior intracerebral haemorrhages (online supplemental table 4). After propensity score matching, 8686 patients remained in the study (4343 (50.0%) treated with apixaban and 4343 (50.0%) treated with warfarin) (figure 1). Balance in baseline characteristics in relation to OAC treatment after propensity score-matched cohort was achieved for most variables (table 1).

**Table 1:** Baseline characteristics in the propensity score-matched cohort

Characteristics	Apixaban (n=4 343)	Warfarin (n=4 343)	P value
Demographics			
Age (years), median (IQR)	71.0 (63.5–77.1)	70.7 (63.3–77.3)	0.95
Sex, male, n (%) (missing)	2 723 (66.2%) [230]	2 844 (66.6%) [75]	0.69
Comorbidities, n (%)			
Diabetes mellitus	797 (18.4%)	797 (18.4%)	1.00
Hypertension	2 558 (58.9%)	2 587 (59.6%)	0.54
Prior stroke (any)	270 (6.2%)	261 (6.0%)	0.72
Prior ischaemic stroke	243 (5.6%)	241 (5.5%)	0.96
Prior unspeciﬁed stroke	15 (0.4%)	15 (0.4%)	1.00
Prior TIA	134 (3.1%)	147 (3.4%)	0.47
COPD	333 (7.7%)	316 (7.3%)	0.51
Asthma	258 (5.9%)	252 (5.8%)	0.82
Heart failure	1 756 (40.4%)	1 787 (41.1%)	0.51
Prior myocardial infarction	888 (20.4%)	913 (21%)	0.53
Prior PCI	457 (10.5%)	487 (11.2%)	0.32
Prior CABG	451 (10.4%)	482 (11.1%)	0.30
Peripheral arterial disease	278 (6.4%)	278 (6.4%)	1.00
Prior systemic embolism	26 (0.6%)	32 (0.7%)	0.51
Prior pulmonary embolism	53 (1.2%)	78 (1.8%)	0.04
Chronic kidney disease	277 (6.4%)	348 (8.0%)	0.004
Renal dialysis	2 (0.1%)	12 (0.3%)	0.02
Liver disease	41 (0.9%)	26 (0.6%)	0.09
Dementia	29 (0.7%)	21 (0.5%)	0.32
Prior major bleeding	429 (9.9%)	430 (9.9%)	1.00
Cancer (within last 3 years)	282 (6.5%)	284 (6.5%)	0.97
CHA_2_DS_2_-VASc score, median (IQR)	3.0 (2.0–4.0)	3.0 (2.0–4.0)	0.30
Apixaban dosage, n (%)			
5.0 mg twice daily	3 691 (85.0%)	–	–
2.5 mg twice daily	652 (15.0%)	–	–
Comedication (within last 6 months), n (%)			
Proton-pump inhibitors	1 409 (32.4%)	1 372 (31.6%)	0.41
ACE inhibitors	1 677 (38.6%)	1 801 (41.5%)	0.01
Angiotensin II antagonists	1 528 (35.2%)	1 466 (33.8%)	0.17
Beta blockers	4 049 (93.2%)	3 858 (88.8%)	<0.0001
Calcium channel antagonists	1 140 (26.2%)	1 140 (26.2%)	1.00
NSAID	267 (6.1%)	194 (4.5%)	0.001
Statins	1 940 (44.7%)	2 068 (47.6%)	0.01
Aspirin	1 098 (25.3%)	1 081 (24.9%)	0.69
Clopidogrel	266 (6.1%)	269 (6.2%)	0.93
Prasugrel	4 (0.1%)	4 (0.1%)	1.00
Ticagrelor	72 (1.7%)	78 (1.8%)	0.68
LMWH	91 (2.1%)	94 (2.2%)	0.88

Continuous variables are presented with medians (IQR) and categorical as numbers (%). Treatment groups compared with Kruskal-Wallis test and χ^2^ test as appropriate. Numbers within square brackets indicate number of missing values.

ACE, angiotensin-converting enzyme; CABG, coronary artery bypass graft; COPD, chronic obstructive pulmonary disease; LMWH, low-molecular-weight heparin; NSAID, non-steroidal anti-inflammatory drug; PCI, percutaneous coronary intervention; TIA, transient ischaemic attack.

### Major bleeding

**Figure 2:**
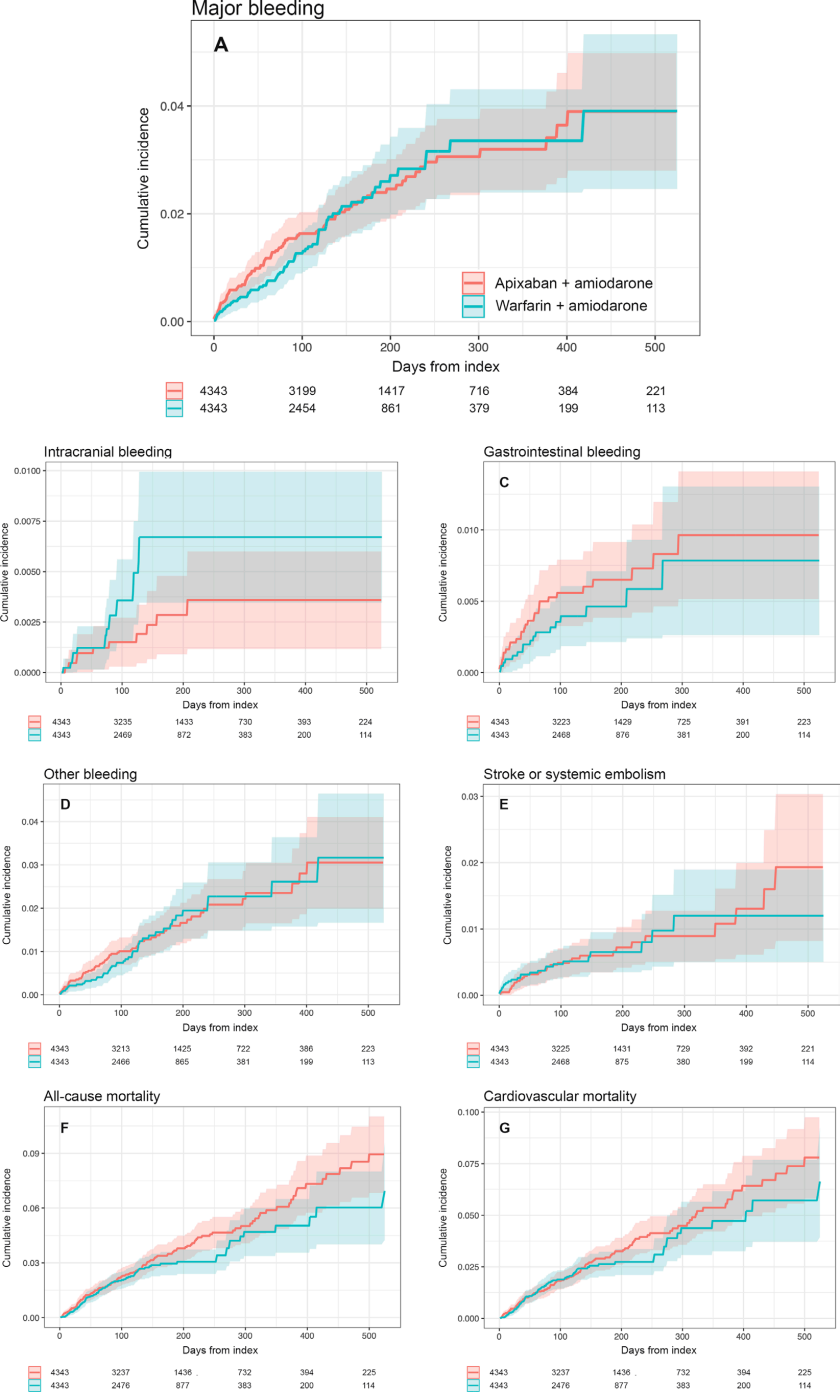
Kaplan-Meier plots for (A) major bleeding, (B) intracranial bleeding, (C) gastrointestinal bleeding, (D) other bleeding (eg, pulmonary or urogenital bleedings), (E) stroke or systemic embolism, (F) all-cause mortality and (G) cardiovascular mortality in the propensity score-matched cohort.

**Figure 3:**
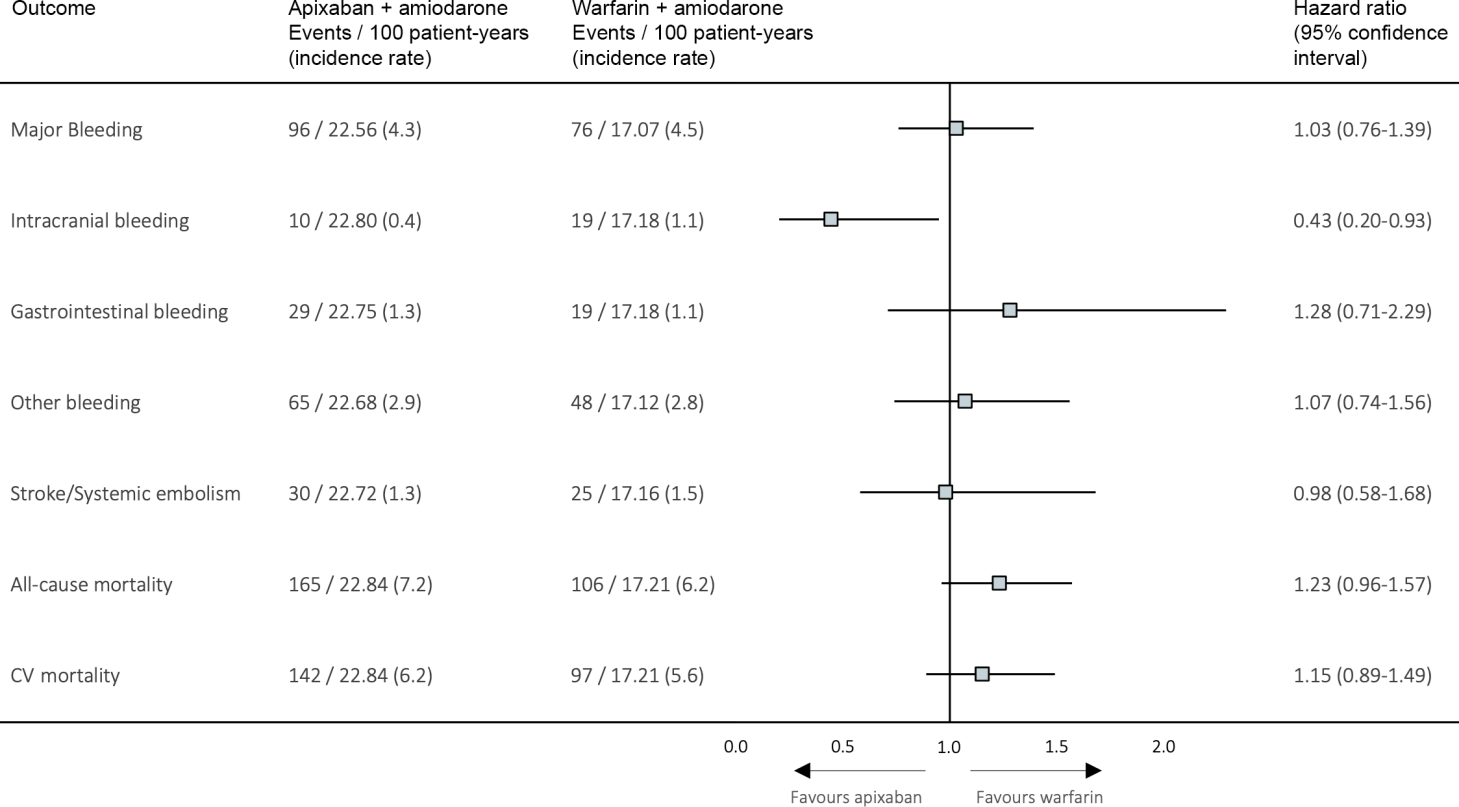
Outcomes in the propensity score-matched cohort. CV, cardiovascular.

Median follow-up was 4.4 months (146 days for the apixaban group and 119 days for the warfarin group), with a total of 172 events for the primary endpoint of major bleeding. Incidence rate of major bleeding events was 4.3/100 patient-years for patients treated with apixaban+amiodarone and 4.5/100 patient-years for patients treated with warfarin+amiodarone (figures 2–3). No statistical difference was observed for major bleeding when comparing apixaban+amiodarone versus warfarin+amiodarone (HR: 1.03; 95% CI: 0.76 to 1.39) (figure 3). For intracranial bleeding, there was a significantly lower risk with apixaban+amiodarone compared with warfarin+amiodarone, although with wide CIs (HR: 0.43; 95% CI: 0.20 to 0.93). No significant differences were found for the other bleeding outcomes, including gastrointestinal bleeding (HR: 1.28; 95% CI: 0.71 to 2.29) and other bleeding events (HR: 1.07; 95% CI: 0.74 to 1.56) (figures 2 and 3).

### Stroke and systemic embolism

The exploratory outcome of stroke and systemic embolism occurred at an incidence rate of 1.3 and 1.5/100 patient-years in the apixaban+amiodarone and the warfarin+amiodarone group, respectively. No significant difference was observed when comparing the treatment strategies (HR: 0.98; 95% CI: 0.58 to 1.68) (figures 2 and 3).

### All-cause mortality and CV mortality

The incidence rate of the exploratory outcome all-cause mortality was 7.2 and 6.2/100 patient-years for patients treated with apixaban+amiodarone versus warfarin+amiodarone, respectively (figures 2 and 3). There was no significant difference in rates of all-cause mortality associated with apixaban+amiodarone compared with warfarin+amiodarone (HR: 1.23; 95% CI: 0.96 to 1.57). The exploratory outcome CV mortality occurred at an incidence rate of 6.2 and 5.6/100 patient-years in the apixaban+amiodarone and the warfarin+amiodarone group, respectively (figures 2 and 3). No significant difference in CV mortality was observed when comparing apixaban+amiodarone versus warfarin+amiodarone (HR: 1.15; 95% CI 0.89 to 1.49).

### Sensitivity analysis

In a sensitivity analysis, only matched apixaban and warfarin-naive patients were included (n=2066). No significant difference was observed for the primary endpoint of major bleeding in the OAC-naive propensity score-matched cohort when comparing apixaban+amiodarone versus warfarin+amiodarone (HR: 1.24; 95% CI: 0.71 to 2.16) (online supplemental figure 1, 2). In the age-related subgroup analysis, including patients aged <75 and ≥75 years, there was no significant difference in major bleeding between the two treatment groups (online supplemental table 5).

## Discussion

This real-world nationwide cohort study, including 8686 propensity score-matched patients showed no significant difference regarding registered major bleeding events in patients with AF treated with amiodarone in combination with apixaban versus warfarin.

Amiodarone is an AAD used in patients with AF and is metabolised in the liver.^3 7^ It is well known that amiodarone interacts with the pharmacokinetics of OACs, including apixaban and warfarin.^8 15^ Thus, it is important to study the benefits and risks associated with different OAC combinations in patients treated with concomitant amiodarone. Our finding of a similar risk of major bleeding associated with apixaban versus warfarin in patients treated with amiodarone aligns with a few prior small observational studies of AAD (amiodarone, sotalol, flecainide, dronedarone, propafenone, dofetilide) and contemporary treatment with apixaban and warfarin.^13 14^ The findings also align with reports from systematic reviews which included data from both observational studies and subgroup analysis of direct oral anticoagulant (DOAC) trials.^16^ However, in a subgroup analysis of the ARISTOTLE trial, specifically comparing apixaban versus warfarin in patients treated with concomitant amiodarone, apixaban was associated with lower rates of major bleeding (HR: 0.61; 95% CI: 0.39 to 0.96).^10^ The differences in the findings from our study and the subgroup analysis from the ARISTOTLE trial might be explained by the difference in study design, the diverse definitions of major bleeding implemented by different studies and the larger sample size and the inclusion of real-life non-selected patients in the current study.

In this study, there was a significant numerically lower rate of intracranial bleeding with apixaban versus warfarin in patients treated with amiodarone. Similar findings have been reported in observational studies of dronedarone with concomitant use of apixaban and warfarin.^17^ Also, the ARISTOTLE trial reported that the risk for intercranial bleeding in patients on apixaban versus warfarin was statically lower with or without amiodarone.^10^ In contrast to the current study, earlier studies have reported higher rates of gastrointestinal bleeding with DOACs other than apixaban compared with warfarin.^16 18 19^ One possible explanation for this disparity could be the lower sensitivity associated with ICD codes for gastrointestinal bleeding (82.6%) compared with other bleeding outcomes.^14^

Another finding in our study was a numerically higher risk of all-cause mortality with apixaban compared with warfarin in patients treated with amiodarone. However, this has not been observed in prior subgroup analysis from DOAC trials or in systematic reviews in which apixaban compared with warfarin in amiodarone-treated patients was associated with an equal or lower risk of all-cause mortality.^10 16 18^ Explanations for this observation could be residual confounding and that patients treated with warfarin in the current study were, despite propensity score matching, more likely to receive concomitant treatment with cardioprotective drugs such as ACE inhibitors and statins, which could partially explain the slightly lower numerical rate of all-cause mortality in warfarin-treated patients.^20 21^

Warfarin has multiple interactions with food and other medication, mainly due to the complete hepatic metabolisation through the CYP enzymes (primarily CYP1A2, CYP2C9 and CYP3A4).^22^ Known interactions between other medications are fewer with apixaban than warfarin, but still present. Apixaban is partly metabolised by the CYP3A4 enzyme but is also eliminated through the P-gp efflux transporters, enabling renal clearance combined with gastrointestinal and hepatobiliary drug excretion.^6^ Amiodarone is a moderate inhibitor of the CYP2C9 and CYP3A4 enzymes and the P-gp transporter with the ability to increase the plasma concentration of both apixaban and warfarin.^6 7 22^ In the ARISTOTLE trial, apixaban was superior to warfarin in preventing stroke or systemic embolism and caused less bleeding.^2^ A more extensive interaction between apixaban and amiodarone may partially explain the findings in the present study, in which apixaban versus warfarin when combined with amiodarone was associated with a similar risk of major bleeding. Still, the findings in the present study suggests that apixaban is a safe OAC in patients with AF treated with amiodarone.

The recommendations from the US FDA and the European EMA include dose reduction recommendations for warfarin in patients who require concomitant amiodarone.^3 7^ While our study confirms the safety of apixaban in patients treated with amiodarone, future studies could explore plasma levels of apixaban in patients treated with amiodarone to fill some knowledge gaps. In clinical practice, the duration and dosage of amiodarone should be kept as short or low as possible to decrease the risk of amiodarone-associated side effects and possible interactions with apixaban.

The main strength of the current study is the large sample size representing real-world nationwide patients with no loss to follow-up. The Swedish national registries also provide unique opportunities to collect data and to combine registries, minimising missing data. To estimate medical therapy over time, the National Prescribed Drug Register was used. However, adherence to prescribed and collected medication cannot be ascertained. Missing data on patient weight prevented the determination of whether the prescribed dosage of apixaban aligned with recommended dose reduction criteria.^3^ Additionally, we lacked information about the true warfarin exposure in terms of INR and TTR. However, in the general warfarin population in Sweden, TTR is high, at around 77.1%.^23^ Due to the observational design of the study no evidence of causation could be established, and the definition of major bleeding was based on ICD-10 codes not defined by the International Society of Thrombosis and Haemostasis (ISTH). Although the definition of major bleeding used in this study has been validated, no clinical event adjudication was made.^14^ Another limitation of the study, potentially influencing the lack of significance for some outcomes, was the short follow-up. Due to the extracardiac toxicity of amiodarone, the treatment duration is often kept short, resulting in most patients only receiving short amiodarone prescriptions. Further limitations include confounding factors (eg, type of AF, use of NSAID) and a potential selection bias despite propensity score matching. Lastly, the primary outcome was a composite endpoint where patients were censored at the time of an event, leaving room for competing risk.

## Conclusions

In real-life patients with AF treated with apixaban versus warfarin and with concomitant use of amiodarone, there were no significant differences in risk of major bleeding. Thus, apixaban and warfarin appear to be equally safe OACs in patients with AF concomitantly treated with amiodarone.

## Data Availability

Data are available upon reasonable request. Restrictions apply to the availability of data, which were used under license and ethical approval and are not publicly available. However, data are available upon reasonable request and with written permission of the Swedish Ethical Review Authority, subject to legal contracts regarding General Data Protection Regulation (GDPR) and Personal Data Processing Agreements.
