# Supplementary material for: Major bleeding in patients with atrial fibrillation treated with apixaban versus warfarin in combination with amiodarone: nationwide cohort study
Source: Open Heart. 2024 Mar 1;11(1):e002555. doi: 10.1136/openhrt-2023-002555 (PMC10910422; doi:10.1136/openhrt-2023-002555)
Supplement: Supplementary data [file openhrt-2023-002555supp001.pdf]

Supplementary Material

Major bleeding in patients with atrial fibrillation treated with apixaban versus warfarin in combination with amiodarone: nationwide cohort study

Contents

Supplementary Table 1. 2
Supplementary Table 2. 3
Supplementary Table 3. 5
Supplementary Table 4. 6
Supplementary Table 5. 8
Supplementary Figure 1. 9
Supplementary Figure 2. 10

Fritz Hansson A, *et al. Open Heart* 2024; 11:e002555. doi: 10.1136/openhrt-2023-002555

Supplementary Table 1. Mean dosage of warfarin/week based on age and sex in Sweden from the Swedish oral anticoagulant registry (AuriculA).

**Mean dosage of warfarin (mg/week) based on age and sex**								
**Age/ Sex**	≤29 years	30-39 years	40-49 years	50-59 years	60-69 years	70-79 years	80-89 years	≥90 years
Male	45.4	50.4	44.5	42.5	36.9	32.0	27.4	24.3
Female	46.7	48.3	44.5	39.4	34.3	28.5	23.7	20.6

Fritz Hansson A, *et al. Open Heart* 2024; 11:e002555. doi: 10.1136/openhrt-2023-002555

Supplementary Table 2. The International Code of Disease, tenth revision (ICD-10) codes applied to identify comorbidities. The Anatomical Therapeutic Chemical (ATC) classification codes applied to identify exposure and co-medications.

Variable	Data Source	ICD-10/ATC code
Exposure		
Amiodarone	National Dispensed Drug Register	C01BD01
Apixaban	National Dispensed Drug Register	B01AF02
Warfarin	National Dispensed Drug Register	B01AA03
Inclusion/exclusion criteria		
Atrial fibrillation	National Patient Register	I48
Mechanical heart valve	National Patient Register	Z952
Mitral stenosis (within last 3 years)	National Patient Register	I342, I050, I052, Q232
Acute venous thrombosis (within last 6 months)	National Patient Register	I26, I801, I802, I803, I808, I809, I822, I823, I828, I829, O223, O871, O882
Hip/knee replacement surgery (within 6 weeks)	National Patient Register	NFB, NFC, NGB, NGC, NFG, NGG
Demographics	National Patient Register	
Age (years), median (IQR)	National Patient Register	
Sex, male	National Patient Register	
Comorbidities	National Patient Register	
Diabetes mellitus	National Patient Register	E10, E11, E12, E13, E14
Hypertension	National Patient Register	I10, I11, I12, I13, I15
Prior stroke (any)	National Patient Register	I60, I61, I63, I64
Prior ischaemic stroke	National Patient Register	I63
Prior unspecified stroke	National Patient Register	I64
Prior TIA	National Patient Register	G458, G459
COPD	National Patient Register	J43, J44
Asthma	National Patient Register	J45, J46
Heart failure	National Patient Register	I42, I50, I110, I255, I130, I132, K761
Prior myocardial infarction	National Patient Register	I21, I22, I23, I252
Prior PCI	National Patient Register	Z955
Prior CABG	National Patient Register	Z951
Peripheral arterial disease	National Patient Register	I70, I71, I72, I73
Prior systemic embolism	National Patient Register	I74
Prior pulmonary embolism	National Patient Register	I26
Chronic kidney disease	National Patient Register	N17, N18
Renal dialysis	National Patient Register	Z992, PBL, PBU, DR016, DR024
Liver disease	National Patient Register	D684C, I850, I859, I982B, K70, K71, K72, K73, K74, K75, K76, K77
Dementia	National Patient Register	F00, F01, F02, F03, F051, G30, G311, G318A
Prior major bleeding	National Patient Register	I60, I61, I62, S064, S065, S066, K226, K250, K252, K254, K256, K260, K262, K264, K266, K270, K272, K274, K276, K280, K282, K284, K286, K290, K625, K661, K920, K921, K922, I850, I983, N02, R319, N939, N950, N501A, H113, H313, H356, H431, H450, H922, I312, J942, M250, R04, R58, T810, D500, D629, T792
Cancer (within last 3 years)	National Patient Register	C
Comedication (within last 6 months)		
Proton pump inhibitors	National Dispensed Drug Register	A02BC
ACE inhibitors	National Dispensed Drug Register	C09A
Angiotensin II antagonists	National Dispensed Drug Register	C09C, C09D
Beta blockers	National Dispensed Drug Register	C07
Calcium channel antagonists	National Dispensed Drug Register	C08
NSAID	National Dispensed Drug Register	M01A (except M01AX05), N02BA01
Statins	National Dispensed Drug Register	C10AA
Aspirin	National Dispensed Drug Register	B01AC06

Fritz Hansson A, *et al. Open Heart* 2024; 11:e002555. doi: 10.1136/openhrt-2023-002555

Clopidogrel	National Dispensed Drug Register	B01AC06
Prasugrel	National Dispensed Drug Register	B01AC04
Ticagrelor	National Dispensed Drug Register	B01AC22
Dalteparin	National Dispensed Drug Register	B01AC24

Supplementary Table 3. The International Code of Disease, tenth revision (ICD-10) codes applied to identify outcomes.

Variable	Data Source	ICD-10/ATC code
Primary outcome		
Major bleeding	National Patient Register<br>National Cause of Death Register	I60, I61, I62, S064, S065, S066, K226, K250, K252, K254, K256, K260, K262, K264, K266, K270, K272, K274, K276, K280, K282, K284, K286, K290, K625, K661, K920, K921, K922, I850, I983, N02, R319, N939, N950, N501A, H113, H313, H356, H431, H450, H922, I312, J942, M250, R04, R58, T810, D500, D629, T792
Secondary outcomes		
Intracranial bleeding	National Patient Register<br>National Cause of Death Register	I60, I61, I62, S064, S065, S066
Gastrointestinal bleeding	National Patient Register<br>National Cause of Death Register	K226, K250, K252, K254, K256, K260, K262, K264, K266, K270, K272, K274, K276, K280, K282, K284, K286, K290, K625, K661, K920, K921, K922, I850, I983
Other bleeding	National Patient Register<br>National Cause of Death Register	N02, R319, N939, N950, N501A, H113, H313, H356, H431, H450, H922, I312, J942, M250, R04, R58, T810, D500, D629, T792
Stroke/systemic thromboembolism	National Patient Register<br>National Cause of Death Register	I63, I74
All-cause mortality	National Cause of Death Register	-
Cardiovascular mortality	National Cause of Death Register	I00-I99

Fritz Hansson A, *et al. Open Heart* 2024; 11:e002555. doi: 10.1136/openhrt-2023-002555

Supplementary Table 4. Baseline characteristics for the study cohort before performing propensity score matching.

Characteristic	Apixaban (n = 4 343)	Warfarin (n = 7 760)
Demographics		
Age (years), median (IQR)	71.0 (63.5 - 77.1)	71.5 (64.5 - 77.9)
Sex, male	2 723 (66.2%) [230]	5 178 (68.1%) [159]
Comorbidities		
Diabetes mellitus	797 (18.4%)	1 472 (19.0%)
Hypertension	2 558 (58.9%)	4 630 (59.7%)
Prior stroke (any)	270 (6.2%)	560 (7.2%)
Prior ischaemic stroke	243 (5.6%)	522 (6.7%)
Prior unspecified stroke	15 (0.35%)	30 (0.4%)
Prior TIA	134 (3.1%)	262 (3.4%)
COPD	333 (7.7%)	516 (6.6%)
Asthma	258 (5.9%)	407 (5.2%)
Heart failure	1 756 (40.4%)	3 344 (43.1%)
Prior myocardial infarction	888 (20.4%)	1 961 (25.3%)
Prior PCI	457 (10.5%)	971 (12.5%)
Prior CABG	451 (10.4%)	960 (12.4%)
Peripheral arterial disease	278 (6.4%)	512 (6.6%)
Prior systemic embolism	26 (0.6%)	60 (0.8%)
Prior pulmonary embolism	53 (1.2%)	143 (1.8%)
Chronic kidney disease	277 (6.4%)	672 (8.7%)
Renal dialysis	2 (0.05%)	23 (0.3%)
Liver disease	41 (0.9%)	42 (0.5%)
Dementia	29 (0.7%)	41 (0.5%)
Prior major bleeding	429 (9.9%)	798 (10.3%)
Cancer (within last 3 years)	282 (6.5%)	433 (5.6%)
CHA_2DS_2-VASc score, median (IQR)	3.0 (2.0 – 4.0)	3.0 (2.0 – 4.0)
Apixaban dosage, n (%)		
5.0 mg twice daily	3 691 (85.0%)	-
2.5 mg twice daily	652 (15.0%)	-
Comedication (within last 6 months)		
Proton pump inhibitors	1 409 (32.4%)	2 426 (31.3%)
ACE inhibitors	1 677 (38.6%)	3 238 (41.7%)
Angiotensin II antagonists	1 528 (35.2%)	2 660 (34.3%)
Beta blockers	4 049 (93.2%)	6 861 (88.4%)
Calcium channel antagonists	1 140 (26.2%)	2 020 (26%)
NSAID	267 (6.1%)	321 (4.1%)
Statins	1 940 (44.7%)	3 824 (49.3%)
Aspirin	1 098 (25.3%)	1 483 (19.1%)
Clopidogrel	266 (6.1%)	425 (5.5%)
Prasugrel	4 (0.1%)	5 (0.1%)
Ticagrelor	72 (1.7%)	104 (1.3%)
LMWH	91 (2.1%)	467 (6%)

Continuous variables are presented with medians (interquartile range [IQR]) and categorical as numbers (%). Treatment groups compared with Kruskal Wallis test and Chi square test as appropriate. Numbers within square brackets indicate number of missing values.

Abbreviations: ACE = angiotensin-converting enzyme. CABG = coronary artery bypass graft. COPD = chronic obstructive pulmonary disease. LMWH = low molecular weight heparin. NSAID = non-steroidal anti-inflammatory drugs. PCI = percutaneous coronary intervention. TIA =transient ischaemic attack.

Supplementary Table 5. Primary outcome in the propensity score matched apixaban and warfarin cohort stratified by age.

Outcome	Hazard ratio (95% confidence interval)	P-value
Major bleeding		
Age < 75 years	0.93 (0.63 – 1.37)	0.71
Age ≥ 75 years	1.23 (0.75 – 1.99)	0.41

Fritz Hansson A, *et al. Open Heart* 2024; 11:e002555. doi: 10.1136/openhrt-2023-002555

Supplementary Figure 1. Kaplan Meier curves for outcomes in matched apixaban and warfarin naïve cohort.

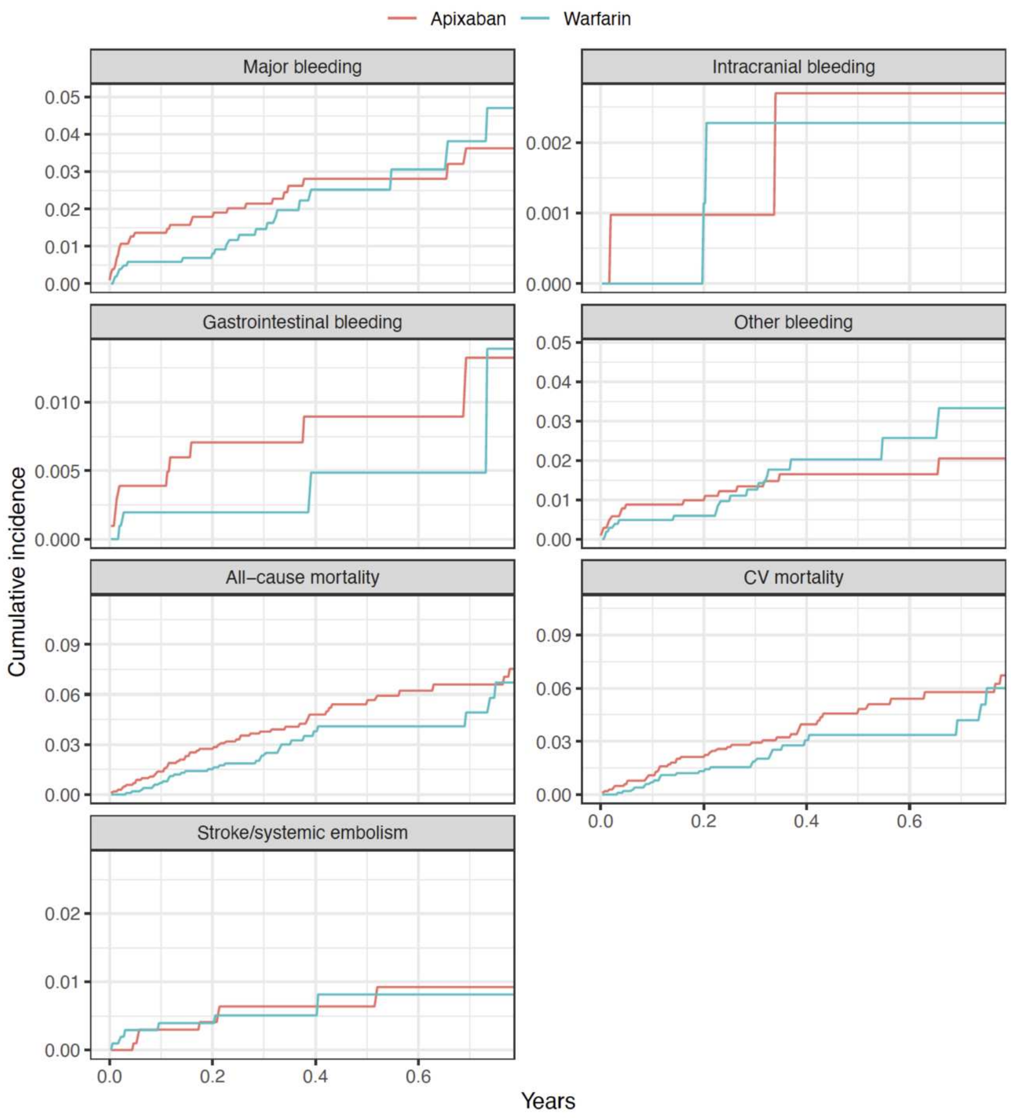

Supplementary Figure 2. Outcomes in the propensity score matched apixaban and warfarin naïve cohort.

Outcome	Apixaban + amiodarone Events / 100 patient-years (incidence rate)	Warfarin + amiodarone Events / 100 patient-years (incidence rate)	Hazard ratio (95% confidence interval)
Major Bleeding	32 / 5.59 (5.7)	21 / 4.18 (5.0)	1.24 (0.71-2.16)
Intracranial bleeding	2 / 5.66 (0.3)	2 / 4.22 (0.5)	0.92 (0.13-6.56)
Gastrointestinal bleeding	11 / 5.63 (2.0)	4 / 4.21 (0.9)	2.11 (0.66-6.74)
Other bleeding	20 / 5.63 (3.6)	18 / 4.19 (4.3)	0.87 (0.46-1.67)
Stroke/Systemic embolism	10 / 5.59 (1.8)	6 / 4.21 (1.4)	1.36 (0.49-3.79)
All-cause mortality	62 / 5.67 (11.0)	36 / 4.22 (8.5)	1.32 (0.87-2.00)
CV mortality	53 / 5.67 (9.3)	31 / 4.22 (7.3)	1.28 (0.82-2.01)

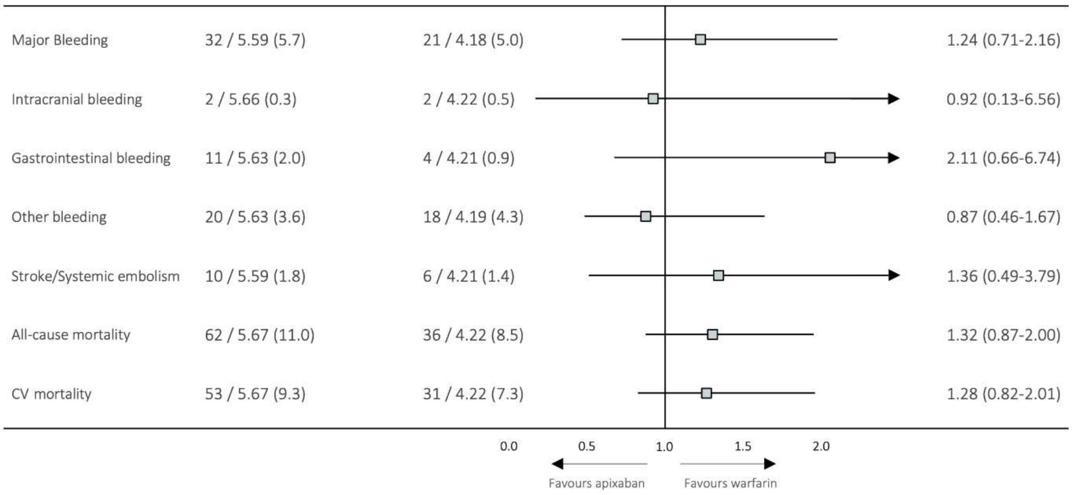

Abbreviations: CV = cardiovascular.

Fritz Hansson A, *et al. Open Heart* 2024; 11:e002555. doi: 10.1136/openhrt-2023-002555
